# New Calibrator with Points Distributed Conical Helically for Online Calibration of C-Arm

**DOI:** 10.3390/s19091989

**Published:** 2019-04-28

**Authors:** Na Guo, Biao Yang, Yuhan Wang, Hongsheng Liu, Lei Hu, Tianmiao Wang

**Affiliations:** School of Mechanical Engineering and Automation, Beihang University, Beijing 100083, China; guona004@buaa.edu.cn (N.G.); ybWipper@buaa.edu.cn (B.Y.); zy1707608@buaa.edu.cn (Y.W.); liuhs@buaa.edu.cn (H.L.); hulei9971@sina.com (L.H.)

**Keywords:** surgical navigation system, online C-arm calibration method, calibration error, ACL reconstruction

## Abstract

To improve the accuracy of calibration of C-arm, and overcome the space limitation in surgery, we proposed a new calibrator for online calibration of C-arm. After the image rectification by a polynomial fitting-based global correction method, the C-arm was assumed as an ideal pinhole model. The relationships between two kinds of spatial calibration errors and the distribution of fiducial points were studied: the performance of FRE (Fiducial Registration Error) and TRE (Target Registration Error) were not consistent, but both were best at the 12 marked points; the TRE decreased with the increase of the uniformity of calibration points distribution, and with the decrease of the distance between the target point and the center of calibration points. A calibrator with 12 fiducial points conical helically distributed, which could be placed on the knee, was an attractive option. A total of 10 experiments on C-arm calibration accuracy were conducted and the mean value of mapping error was 0.41 mm. We designed an ACL reconstruction navigation system and carried out specimen experiments on 4 pairs of dry femur and tibia. The mean accuracy of navigation system was 0.85 mm, which is important to the tunnel positioning for ACL reconstruction.

## 1. Introduction

Preservation of remnants of cruciate ligament in ACL (anterior cruciate ligament) reconstruction, which could promote vascular growth of the transplant [1], has now been common in clinical trials. The accurate location of the femoral and tibial tunnels was the most conducive factor for the recovery of ligaments proprioception [2,3,4]. But ligament remnants preservation would make accurate positioning of femoral and tibial tunnels difficult [5]. As bone tissues could be displayed clearly in X-Ray images or computed tomography (CT) images [6], a fluoroscopy navigation based on C-arm might be an optional method for ACL reconstruction navigation system. It could improve accuracy of femoral and tibial tunnels placement, and help trainee-surgeons to relieve fatigue and accelerate the learning curve. Multi-spaces are included in the navigation system, such as X-ray image space, visual space and instrument space. All spaces should be unified in visual coordinate system. The calibration of C-arm, which is to establish the mapping between surgical space and X-ray image space (captured by C-arm), is primary for surgical navigation system to improve the precision of the positioning of surgical path [6,7,8,9].

Since the first application of navigation technology in 1980s, the calibration technology of C-arm, getting 3D information from 2D images [10], has drawn much attention [11]. Image distortion correction, targets recognition, calibration models of C-arm and design of calibration phantom are the research focuses [12,13,14,15,16,17,18,19,20,21,22,23,24,25,26,27,28,29,30,31,32,33]. There were three types of geometric distortion in the process of X-ray imaging [12,13,14]: pincushion distortion, sigmoidal distortion and local distortions. To correct the image distortion for the accurate calibration of C-arm, local correction method and global correction method were proposed [10]. The raw images with distortion were divided into multiple parts in local method, and these sub-images were corrected separately. Larger numbers of reference points were required, which limited surgical vision and caused complex calculations. Besides, the local method could not avoid the boundary distortions. In fact, the polynomial global correction method was commonly implanted in a navigation system [15,16,17,18]. Recognition accuracy of reference points for image rectification would have an impact on the accuracy of calibration of C-arm [19]. Besides, it would be time consuming to recognize reference points manually. Reference points for image rectification should be recognized automatically. Luan Sheng [10] proposed a gray scale weighted centroid method to extract points; Xiaojun Zhou [15] recognized points through morphology; Zhang Jianfa [17] separated points from background by automatic threshold method and calculated the centroid and area of the projection of small balls by Connected Components (CC) method. Previous studies proposed pinhole model [15,18] and nonlinear model [19] for the calibration models of C-arm. The nonlinear model, which considered the image distortion, required a large number of markers and complex calculation for high calibration accuracy. Thus, pinhole model may be more proper for online system with simple computation [20,21,22]. This paper took the C-arm as an ideal pinhole model, with X-ray images accurately corrected.

A calibrator with fiducial points was necessary in the navigation system based on C-arm [18]. For specific surgical demands, literature [21,22] developed a pose determination system 2 (PDS2), and references [15,16,17,19,23,24] adopted a biplane calibrator. There were 108 fiducial points placed around a cylinder with a diameter of 14 cm along the z-axis of PDS2. The PDS2 was used to calibrate the C-arm offline with complex calculation [21,22]. Steel balls, the fiducial points, could have an impact on the gray distribution of X-ray images. This will jeopardize registration success in surgical navigation system, especially using intensity-based 2D-3D registration. If there were too many fiducial points, they would cover each other, which would make recognition of them difficult. Besides, targets might be covered by the fiducial points in X-Ray images. The number of fiducial points should not be too large. A biplane calibrator was utilized to build the relationship between the images and their coordinates in the world online [15,19]. The number of fiducial points of a biplane calibrator was less than 12. The position and attitude of visual tracking sensor might be moved in different navigation procedures, if a calibrator was at the end of image receiver of C-arm. The adjustment of visual sensor is inconvenient in surgery. The accuracy of space registration might be better when the targets were near the space of the registration points than far away from that. All biplane calibrators were installed at the X-ray image intensifier, with surgical targets far away from the calibration phantom in their navigation system. This would cause the matching of X-ray images and 3D space inaccuracy. 

We proposed a new type of calibration phantom, which could be placed on the knee joint. Surgical targets were in the space of the calibrator. The number or the distribution of calibration points had an impact on the errors of spatial registration [25,28]. We studied the relationships between kinds of spatial calibration errors and the distribution of fiducial points. A calibrator with 12 points uniformly distributed on a conical spiral curve was designed in this paper. It was applied in a navigation and positioning system for ACL reconstruction.

## 2. Online C-Arm Calibration Method for Surgery Navigation System

### 2.1. Image Distortion Correction

**Figure 1:**
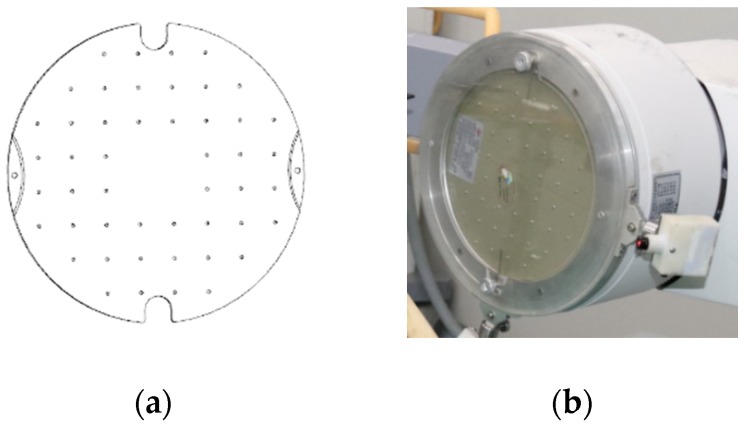
Rectification plate: (**a**) a rectification plate model; (**b**) Plate installment.

**Figure 2:**
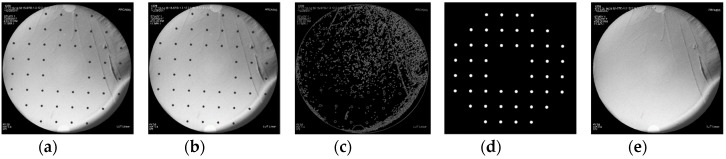
Procedures of image distortion correction: (**a**) Raw image; (**b**) Median filter; (**c**) Edge detection; (**d**) Target Recognition; (**e**) Inpainting image.

To implement a polynomial fitting-based global correction method, we designed a rectification plate (Figure 1a). The plate was made of Perspex, and it was fixed to a steel ring by two screws. The ring was installed at the X-ray image intensifier (Figure 1b) by four screws. 48 steel balls were uniformly distributed on the plate, with a diameter of 2.5 mm and an interval of 25 mm (Figure 1a). The centers of steel balls represented the reference points. The projections of steel balls were clear on X-ray image (Figure 2a). 

There were four steps in the process of the image distortion rectification using a global polynomials:

(1) Reference points were recognized automatically. 

The recognition of reference points was implemented as follows: firstly, the raw X-ray image (Figure 2a) was filtered with a Median filter (Figure 2b); secondly, Canny operator was used to segment the edge of image (Figure 2c); thirdly, Hough transform was adopted to identify the center and radius positions of the reference points (Figure 2d) [29]. The dynamic linked list structure was used to allocate cumulative parameters to reduce the memory demand. Figure 2d was generated by computer. The background of Figure 2d was black, and the size of it was the same as that of the raw image. Steel balls in Figure 2d were in white color. 

(2) Correction factors were calculated.

Centers of the 48 steel balls $P_{i}(i=1,2,\ldots ,48)$ in ideal image in template and X-ray images were used to calculate the correction factors. $\left(x_{i},y_{i}\right)$ were coordinates of $P_{i}$ in ideal image, which were determined by the design of rectification plate. $\left(u_{i},v_{i}\right)$ were coordinates of $P_{i}$ in raw image, which could be calculated by methods in Step 1). If $a_{j}$ and $b_{j}(j=0,1,2,3,4,5)$ represented correction factors, the relationship between $\left(x_{i},y_{i}\right)$ and $\left(u_{i},v_{i}\right)$ could be expressed as follows:

(1) $$\left[\begin{array}{c} u_{i}\\ v_{i} \end{array}\right]=\left[\begin{array}{cccccc} a_{0} & a_{1} & a_{2} & a_{3} & a_{4} & a_{5}\\ b_{0} & b_{1} & b_{2} & b_{3} & b_{4} & b_{5} \end{array}\right]\times S_{i}$$

where $S_{i}={\left[\begin{array}{cccccc} 1 & x_{i} & y_{i} & x_{i}{}^{2} & y_{i}{}^{2} & x_{i}y_{i} \end{array}\right]}^{T}$ (i=1,2,3,…,n).

If the number of rectified reference points was n, there were 2n equations according to Equation (1) [15]:

(2) $$\left[\begin{array}{ccc} u_{1} & & v_{1}\\ & \vdots & \\ u_{n} & & v_{n} \end{array}\right]=\left[\begin{array}{c} S_{1}{}^{T}\\ \vdots \\ S_{n}{}^{T} \end{array}\right]\times {\left[\begin{array}{cccccc} a_{0} & a_{1} & a_{2} & a_{3} & a_{4} & a_{5}\\ b_{0} & b_{1} & b_{2} & b_{3} & b_{4} & b_{5} \end{array}\right]}^{T}$$

$a_{j}$ and $b_{j}$ were calculated by least square method according to Equation (2).

(3) Gray values of all pixels of rectified image were computed.

If g(x,y) represented gray value of each pixel (x,y) in rectified image, the position of (x,y) in raw image would be $\left(x^{\prime },y^{\prime }\right)$ according to Equation (1). The value of g(x,y) was equal to the gray value $f(x^{\prime },y^{\prime })$ of $\left(x^{\prime },y^{\prime }\right)$ in raw image. If 4 integer pixels close to $\left(x^{\prime },y^{\prime }\right)$ were $\left(x_{0},y_{0}\right)$, $\left(x_{0},y_{1}\right)$, $\left(x_{1},y_{0}\right)$, $\left(x_{1},y_{1}\right)$, and gray values of them in the raw image were $f_{00}$, $f_{01}$, $f_{10}$, $f_{11}$, the gray value $f(x^{\prime },y^{\prime })$ could be calculated by a bilinear interpolation algorithm as follows:

(3) $$f(x^{\prime },y^{\prime })=[x_{1}-x\prime ,x^{\prime }-x_{0}]\left[\begin{array}{cc} f_{00} & f_{01}\\ f_{10} & f_{11} \end{array}\right]\left[\begin{array}{c} y_{1}-y^{\prime }\\ y^{\prime }-y_{0} \end{array}\right]$$

where, $x_{1}-x_{0}=1$ and $y_{1}-y_{0}=1$.

Gray values g(x,y) of all pixels in rectified image were computed according to Equation (3).

(4) Reference points were hidden through an inpainting algorithm.

To eliminate the visual disturbance caused by reference points, an image inpainting technique based on the fast marching method (FMM) [26] was applied. We inpainted pixels from the outside of the circles in Figure 2b to the inside. Figure 2e was the result of Figure 2b after inpainting.

### 2.2. Pinhole Model

• An ideal pinhole model

**Figure 3:**
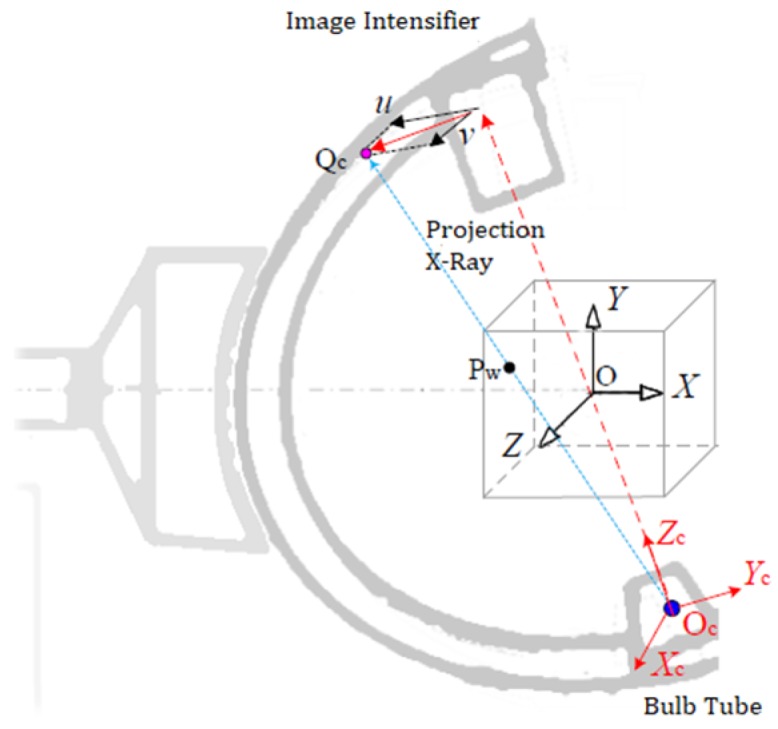
Imaging principle of c-arm.

After rectification of image distortion, the C-arm can be considered an ideal pinhole model. If the coordinate of the point $P_{w}$ (Figure 3) in world coordinate system was (x,y,z), the coordinate of it in C-arm coordinate system was $\left(x_{c},y_{c},z_{c}\right)$, and the pixel coordinate of it in X-ray image was (u,v), the relationship between different coordinates could be expressed as follows.

(4) s[uv1]=[fdx0u000fdyv000010][RT01][xyz1]

(5) $$R=\left[\begin{array}{ccc} r_{11} & r_{12} & r_{13}\\ r_{21} & r_{22} & r_{23}\\ r_{31} & r_{32} & r_{33} \end{array}\right]=\left[\begin{array}{ccc} C\beta C\gamma & S\alpha S\beta C\gamma +C\alpha S\gamma & -C\alpha S\beta C\gamma +S\beta S\gamma \\ -C\beta S\gamma & -S\alpha S\beta S\gamma +C\alpha C\gamma & C\alpha S\beta S\gamma +S\alpha C\gamma \\ S\beta & -S\alpha C\beta & C\alpha C\beta \end{array}\right]$$

where, C represented cos, S represented sin. Five intrinsic parameters were as follows: f represented focal length of C-arm, dx and dy represented pixel size, $\left(u_{0},v_{0}\right)$ represented the position of focus of C-arm in the X-Ray image; six extrinsic parameters were as follows: $T={\left[t_{x},t_{y},t_{z}\right]}^{T}$ represented translation vectors between world coordinate system and C-arm coordinate system, R represented rotation matrix between world coordinate system and C-arm coordinate system,(α,β,γ) represented three Euler angles. 

If $k_{1}=f/dx$, $k_{2}=f/dy$, $A_{1}=\frac{k_{1}r_{11}+r_{31}u_{0}}{t_{z}}$, $A_{2}=\frac{k_{1}r_{12}+u_{0}r_{32}}{t_{z}}$, $A_{3}=\frac{k_{1}r_{13}+u_{0}r_{33}}{t_{z}}$, $A_{4}=\frac{k_{1}t_{x}+u_{0}t_{z}}{t_{z}}$, $A_{5}=\frac{r_{31}}{t_{z}}$, $A_{6}=\frac{r_{32}}{t_{z}}$, $A_{7}=\frac{r_{33}}{t_{z}}$, $A_{8}=\frac{k_{2}r_{21}+r_{31}v_{0}}{t_{z}}$, $A_{9}=\frac{k_{2}r_{22}+v_{0}r_{32}}{t_{z}}$, $A_{10}=\frac{k_{2}r_{23}+v_{0}r_{33}}{t_{z}}$, and $A_{11}=\frac{k_{2}t_{y}+v_{0}t_{z}}{t_{z}}$, Equation (6) could be deduced from Equation (4):

(6) $$\begin{array}{l} A_{1}x+A_{2}y+A_{3}z+A_{4}-A_{5}xu-A_{6}yu-A_{7}zu=u\\ A_{8}x+A_{9}y+A_{10}z+A_{11}-A_{5}xv-A_{6}yv-A_{7}zv=v \end{array}$$

If the coordinates of n(n≥6) fiducial points in world coordinate and in the X-ray image were known as $\left(x_{i},y_{i},z_{i}\right)$ and $\left(u_{i},v_{i}\right)$ (i=1,2,⋯n), there were 2n linear equations about $A_{i}(i=1,2,\cdots 11)$, which could be expressed as follows:

(7) E⋅A=C

where,

$E_{(2\times n)\times 11}={\left[\begin{array}{ccccccccccc} x_{1} & y_{1} & z_{1} & 1 & -x_{1}u_{1} & -y_{1}u_{1} & -z_{1}u_{1} & 0 & 0 & 0 & 0\\ & \vdots & \vdots & & & \vdots & \vdots & & & \vdots & \\ x_{n} & y_{n} & z_{n} & 1 & -x_{n}u_{n} & -y_{n}u_{n} & -z_{n}u_{n} & 0 & 0 & 0 & 0\\ 0 & 0 & 0 & 0 & -x_{1}v_{1} & -y_{1}v_{1} & -z_{1}v_{1} & x_{1} & y_{1} & z_{1} & 1\\ & \vdots & \vdots & & & \vdots & \vdots & & & \vdots & \\ 0 & 0 & 0 & 0 & -x_{n}v_{n} & -y_{n}v_{n} & -z_{n}v_{n} & x_{n} & y_{n} & z_{n} & 1 \end{array}\right]}_{(2\times n)\times 11}$
$A_{11\times 1}={\left[\begin{array}{ccccccccccc} A_{1} & A_{2} & A_{3} & A_{4} & A_{5} & A_{6} & A_{7} & A_{8} & A_{9} & A_{10} & A_{11} \end{array}\right]}^{T}$
$C_{(2\times n)\times 1}={\left[\begin{array}{cccccccc} u_{1} & u_{2} & \cdots & u_{n} & v_{1} & v_{2} & \cdots & v_{n} \end{array}\right]}^{T}$

$A_{i}$ could be calculated according to the equation $A={\left(E^{T}E\right)}^{-1}E^{T}C$ by least square method according to direct linear transform (DLT) [27] and be used in surgical navigation system. 

• A back projection model

If (u,v) represented the coordinates of point P in X-ray images, the equation of space line l corresponding to P could be expressed as follows:

(8) $$\left[\begin{array}{ccc} A_{5}u-A_{1} & A_{6}u-A_{2} & A_{7}u-A_{3}\\ A_{5}v-A_{8} & A_{6}v-A_{9} & A_{7}v-A_{10} \end{array}\right]\left[\begin{array}{c} x\\ y\\ z \end{array}\right]=\left[\begin{array}{c} u-A_{4}\\ v-A_{11} \end{array}\right]$$

If $l_{a}$ and $l_{l}$ were space lines corresponding to $P_{a}(u_{a},v_{a})$ and $P_{l}(u_{l},v_{l})$ respectively, and $P_{a}$ and $P_{l}$ in X-ray images were the projection points of the same space point P at anteroposterior and lateral projection respectively, the intersection of $l_{a}$ and $l_{l}$ would be the world coordinates of P. In fact, it was possible that space lines $l_{a}$ and $l_{l}$ might not intersect. The world coordinates of P should be calculated by least square method.

### 2.3. Design of Online C-Arm Calibrator

#### 2.3.1. Conical Spiral Curve Model for C-arm Calibration

For fast and accurate calibration, calibrator under the following conditions would be preferred: (1) the knee joint, the area of the targets should be within the space of the calibrator; (2) numbers of markers on the calibrator should be less than 20, and 6 markers at least should be imaged on the X-ray image at different projection directions, especially at anteroposterior and lateral projection; (3) markers should avoid blocking each other. We took conical spiral curve as a model of markers on calibration phantom.

(9) $$\begin{array}{l} x=\mathrm{rr}\cdot \exp(-t/pp)\cdot \cos(t)\\ y=\mathrm{rr}\cdot \exp(-t/pp)\cdot \sin(t)\\ z=h\cdot t \end{array}$$

**Figure 4:**
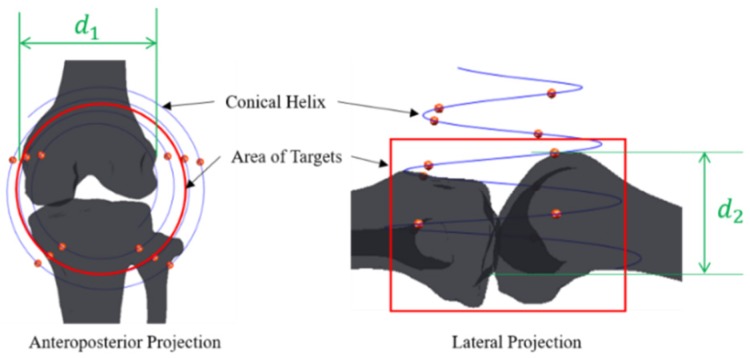
A conical spiral curve model of markers on calibration phantom.

Since convex patella was above the knee joint, the calibration phantom based on conical helix could be placed on the knee joint. The tunnels for ACL reconstruction were in the space of the calibrator. Actual spaces of 10 knee joints were measured on 3-D reconstruction models from CT data to provide reference for the model of conical spiral curve (Figure 4). $d_{1}$ was maximum distance between lateral femoral wall and medial femoral condyle, and $d_{2}$ was maximum distance between top of knee joint and resident ridge at a flexion angle of 90° to 150°. The value of $d_{1}$ ranged from 64.58 to 68.89, and the value of $d_{2}$ ranged from 55.34 to 59.02. Taking soft tissue into account, the parameters of truncated cone were designed as follows: diameter of large circle at the bottom of truncated cone was 120 mm, which was about twice as much as $d_{1}$; diameter of small circle at the top of truncated cone was 60 mm, which was equal to $d_{1}$; height of truncated cone between the bottom and the top was 87.5 mm, which was about 1.5 times as much as $d_{2}$. Fiducial points might cover each other, if the number of spiral circles was too many. There were 3.5 circle on conical spiral curve, with a pitch of 25 mm. The parameters of conical spiral curve were as follows: maximum radius rr=60, convergence rate pp=31.529, rise rate h=3.979, and variables t=(0,7π).

#### 2.3.2. Definitions of Errors of Point Matching Spatial Registration

There were three kinds of errors defined in point matching [28,30]: fiducial localization error (FLE), fiducial registration error (FRE), and target registration error (TRE). The number and the distribution of fiducial points would have an influence on the accuracy of the target registration [25,31,32,33]. N. M. Hamming [31] studied target registration errors of four phantoms with different distributions of markers. Manning Wang [32] classified all the errors in neurosurgery navigation system into two groups, and proposed a method with distribution templates of the fiducial points. FLE, FRE and TRE in the calibration of C-arm were defined as follows:

• Fiducial Localization Error

Fiducial localization error (FLE) was the primary cause of FRE and TRE. In calibration of C-arm, FLE resulted from image distortion correction errors, fiducial point extraction errors and other errors. It was the average error between the coordinates of fiducial points in ideal image and in real image, and would be defined as follows:

(10) $$FLE=\frac{1}{n}\sum_{i=1}^{n}\sqrt{{\left[u_{i}-(u_{i}+\Delta u_{i})\right]}^{2}+{\left[v_{i}-(v_{i}+\Delta v_{i})\right]}^{2}}$$

where, the ideal coordinates of fiducial points $P_{i}$(i=1,2,…,n) in 2D images was $\left(u_{i},v_{i}\right)$, with errors $\left(\Delta u_{i},\Delta v_{i}\right)$ during the calibration of C-arm.

• Fiducial Registration Error

Fiducial registration error (FRE) was the average error between the real position of fiducial points and the calculated position of them through a back projection model in Section 2.2. It could be defined as follows:

(11) $$FRE=\frac{1}{n}\sum_{i=1}^{n}\sqrt{{\left({x^{\prime }}_{i}-x_{i}^{0}\right)}^{2}+{\left({y^{\prime }}_{i}-y_{i}^{0}\right)}^{2}+{\left({z^{\prime }}_{i}-z_{i}^{0}\right)}^{2}}$$

where, $\left(x_{{}_{i}}^{0},y_{{}_{i}}^{0},z_{{}_{i}}^{0}\right)$ represented the real world coordinates of fiducial points $P_{i}$, $\left({x^{\prime }}_{i},{y^{\prime }}_{i},{z^{\prime }}_{i}\right)$ represented the calculated coordinates of $P_{i}$ by a back projection model.

• Target Registration Error

Target registration error (TRE) was the average error between the real position of targets and the calculated position of them through a back projection model in Section 2.2. It could be defined as follows:

(12) $$TRE=\frac{1}{N}\sum_{i=1}^{N}\sqrt{{\left(x_{i}^{t}{}^{\prime }-x_{i}^{t}\right)}^{2}+{\left(y_{i}^{t}{}^{\prime }-y_{i}^{t}\right)}^{2}+{\left(z_{i}^{t}{}^{\prime }-z_{i}^{t}\right)}^{2}}$$

where, $\left(x_{i}^{t},y_{i}^{t},z_{i}^{t}\right)$ represented the real world coordinates of targets, $\left({x^{\prime }}_{i},{y^{\prime }}_{i},{z^{\prime }}_{i}\right)$ represented the calculated coordinates of targets by a back projection model.

TRE was the most objective measure of registration accuracy. However, it should be estimated through algorithms and could not be measured directly and accurately in surgery. FRE, which was easy to estimate, was most commonly used in point-based registration algorithm.

#### 2.3.3. Relationship between Fiducial Points and Registration Errors

Taking consideration of C-arm commonly used in orthopedics operation, the parameters of pinhole model were assumed as follows: a Gaussian model of FLE $\left(\Delta u_{i},\Delta v_{i}\right)$~N(0,2.34); pixel coordinates of the center of the X-ray image $\left[u_{0},v_{0}]=[512,512\right]$, focal distance f=900, pixel scales [dx,dy]=[0.15,0.15]; at anteroposterior projection, rotation parameter $\left[\alpha_{A},\beta_{A},\gamma_{A}]=[0,0,0\right]$, translation parameters $\left[x_{A},y_{A},z_{A}]=[0,0,800\right]$; at lateral projection, rotation parameter $\left[\alpha_{L},\beta_{L},\gamma_{L}]=[90,0,0\right]$, translation parameters $\left[x_{L},y_{L},z_{L}]=[0,0,800\right]$.

1. Relationship between number of fiducial points and spatial calibration errors 

**Figure 5:**
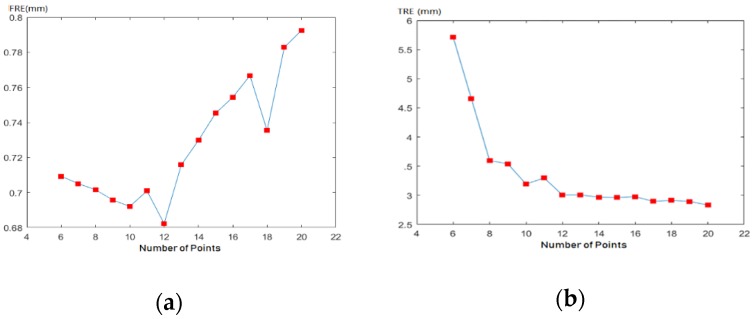
Relationship between number of fiducial points and spatial calibration errors: (**a**) relationship between number of fiducial points and FRE; (**b**) relationship between number of fiducial points and TRE.

Under different number of points, 10000 groups of points were randomly generated on the conical spiral curve. The numbers of points extended from 6 to 12. FRE and TRE were calculated at different numbers of points respectively. The mean values of FRE and TRE of 10000 groups were calculated. Figure 5a showed that FRE decreased with the increase of the number of points when the number was less than 12, but increased with the increase of the number when it was more than 12. However, the performance of FRE and TRE were not consistent in Figure 5. TRE decreased with the increase of the number of points in general, but was stabilized over 12 in Figure 5b. As the increase of the number of points would produce additional unwanted shade in X-images, 12 points would be preferred for calibration of C-arm.

2. Relationship between uniformity of a points set and TRE

Uniformity of finite point set is a measure of point set pattern, which describes the spatial relationship in point set [34]. If numbers of points in different region or direction are equal or similar to each other, the points set is well-uniformly distributed. Otherwise, if points are concentrated on one or several regions, which means that number of points varies widely in different region or direction, the points set is unevenly distributed. If the relationship of a point set $A=\left\{p_{1},p_{2},\cdots ,p_{n}\right\}$ and a 3-dimensional space C was A∈C, then uniformity of A in C was defined as follows [35]:

(13) $$E(A)=\frac{\underset{q\in C}{\sup}\underset{1\leq i\leq n}{\min}d(q,p_{i})}{R}$$

where, $\bar{p}=\frac{1}{n}\sum_{i=1}^{n}p_{i}$ represented the center of a point set A, $R=\sqrt{\frac{1}{n-1}\sum_{i=1}^{n}d(x_{i},\bar{x})}$ represented the standard deviation of A and $d^{2}(q,p)={\left(q-p\right)}^{\prime }(q-p)$. d(q,p) represented Euclidean distance between q and p. If q∈C, $d_{q}=\underset{q\in C}{\sup}\underset{1\leq i\leq n}{\min}d(q,p_{i})$ is called supremum-minimum distance of A. $d_{q}$ was the supremum radius of open circle subsets of C without A.

**Figure 6:**
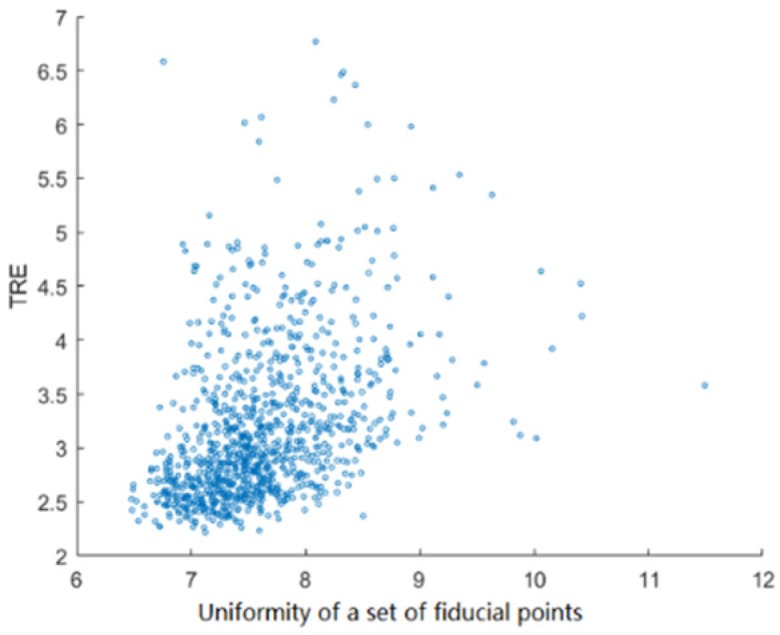
Relationship between uniformity of a points set and TRE.

The surgical space was assumed as $C\in \{(x,y,z)|x^{2}+y^{2}+z^{2}\leq 100^{2}\}$. Number of calibrator points is 12. There were N targets in the surgical space C. The intervals of targets and q were certain. Multi groups of points were randomly generated on the conical spiral curve in Figure 6. Uniformity and TRE of each group were calculated. Pearson Correlation Coefficient between the uniformity of point set and TRE was 0.4224. Overall, TRE and E(A) were neutral positive related.

3. Relationship between spatial location relations of targets to calibrator and TRE

**Figure 7:**
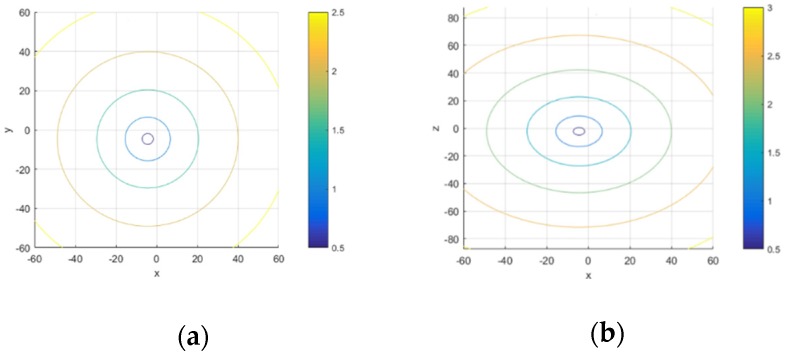
Relationship between location of target with calibrator and TRE: (**a**) Isopleth of TRE at projection of XOY plane; (**b**) Isopleth of TRE at projection of XOZ plane.

A group of 12 fiducial points were randomly generated on the conical spiral curve. Target points were at the planes which are parallel to plane XOY and plane XOZ. The center of calibrator points were considered as origin of the planes. Target points were sampled on the planes with a TRE interval of 0.5. TRE increased with the increase of distance between targets and marker points in Figure 7.

#### 2.3.4. Design of Online C-arm Calibrator

**Figure 8:**
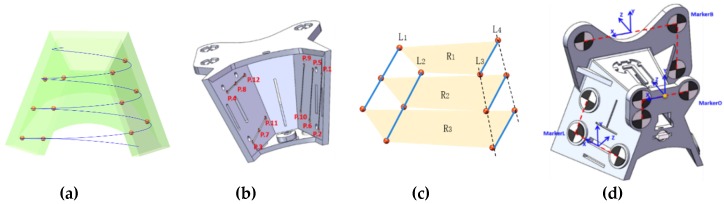
Design of online C-arm calibrator: (**a**) design model of calibrator; (**b**) distribution of 12 fiducial points; (**c**) projection model of fiducial points; (**d**) distribution of visual markers.

A calibrator with 12 points uniformly distributed on the conical spiral curve (UDCSC) could provide better accuracy. Intersection points of the conical spiral curve and two planes were taken as fiducial points for calibration in Figure 8a. Twelve steel balls with a diameter of 4 mm were embedded in the calibrator, with the following relations (Figure 8b,c): (1) all 12 markers were on the conical spiral curve; (2) points of $P_{1},P_{2},P_{5},P_{6},P_{9},P_{10}$ were on one plane, and points of $P_{3},P_{4},P_{7},P_{8},P_{11},P_{12}$ were on another plane; (3) points of $P_{1},P_{5},P_{9}$, points of $P_{2},P_{6},P_{10}$,points of $P_{3},P_{7},P_{11}$, or points of $P_{4},P_{8},P_{12}$ were collinear; (4) there were grooves at lines through points of $P_{3},P_{7},P_{11}$, points of $P_{4},P_{8},P_{12}$, points of $P_{1},P_{2}$, points of $P_{5},P_{6}$ and points of $P_{9},P_{10}$; (5) points of $P_{1},P_{2},P_{3},P_{4}$, points of $P_{5},P_{6},P_{7},P_{8}$ and points of $P_{9},P_{10},P_{11},P_{12}$ were vertexes of convex quadrilaterals on the images of different projections. Four visual markers were distributed on the four planes of the calibrator for navigation. Each consisted of three black-white circles.

## 3. Experiments and Results

### 3.1. Experiment of Rectification Error

The cross points of the grid on the test plate were recognized automatically. The pixel coordinates and physical coordinates of the crosses were $\left(u_{i},v_{i}\right)$ and $\left(x_{i},y_{i},1\right)$, the relationship between them could be expressed as follows:

(14) $$\left[\begin{array}{c} u_{i}\\ v_{i} \end{array}\right]=[\begin{array}{cc} sR & t \end{array}]\left[\begin{array}{c} x_{i}\\ y_{i}\\ 1 \end{array}\right]$$

where, R represented rotation matrices, t represented offset, s represented scaling factor.

Distortion at the center of X-image was smallest. Nine points in the center of the plate were used to calculate the similarity transformation matrix $\left[\begin{array}{cc} sR & t \end{array}\right]$. The ideal position of all cross points could be calculated by Equation (14). If $\left(u_{i}^{I},v_{i}^{I}\right)$ represented ideal position of cross points, (uiRec,viRec) represented pixel position of cross points in rectified image, and $\left(u_{i}^{\mathrm{Raw}},v_{i}^{\mathrm{Raw}}\right)$ represented pixel position of cross points in raw image, the error between ideal position and pixel position of cross points in rectified image ${\mathrm{Err}}_{i}^{\mathrm{Rec}}$ and the error between ideal position and pixel position of cross points in raw image ${\mathrm{Err}}_{i}^{\mathrm{Raw}}$ could be expressed as follows:

(15) $$\begin{array}{l} {\mathrm{Err}}_{i}^{\mathrm{Rec}}=\sqrt{{\left(u_{i}^{I}-u_{i}^{\mathrm{Rec}}\right)}^{2}+{\left(v_{i}^{I}-v_{i}^{\mathrm{Rec}}\right)}^{2}}\\ {\mathrm{Err}}_{i}^{\mathrm{Raw}}=\sqrt{{\left(u_{i}^{I}-u_{i}^{\mathrm{Raw}}\right)}^{2}+{\left(v_{i}^{I}-v_{i}^{\mathrm{Raw}}\right)}^{2}} \end{array}$$

**Figure 9:**
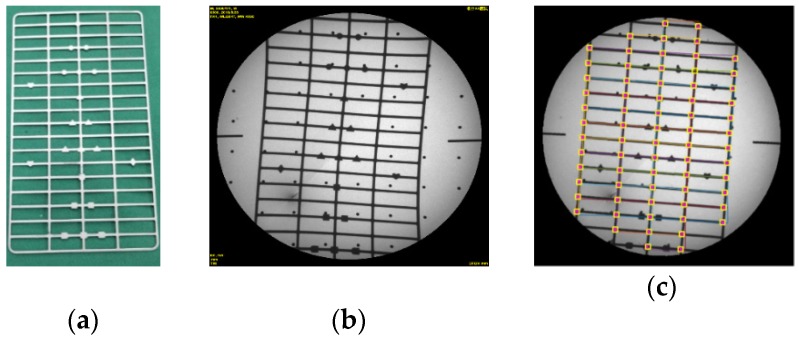
Image rectification test: (**a**) Test grid plate; (**b**) Raw X-Ray image of test plate; (**c**) Rectified Images. “×” represented the position of the grid points of the test plate in the raw image, “——” represented the grid distribution of the test plate in the raw image and □ represented the ideal position of the test plate.

**Figure 10:**
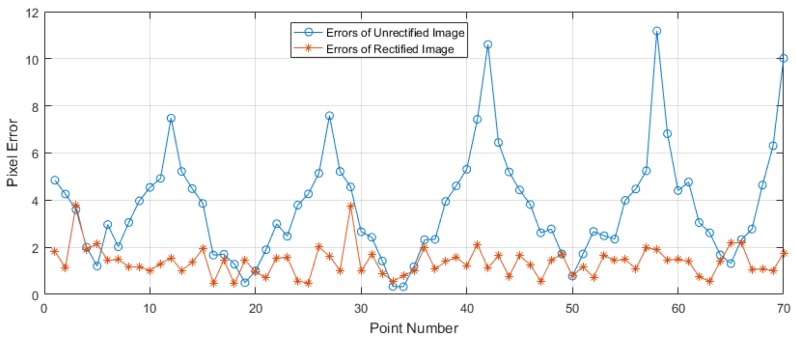
Comparison of the errors of rectified and unrectified X-ray image, at the vertical dimension between test plate and receiving end of C-arm of 45 mm.

**Figure 11:**
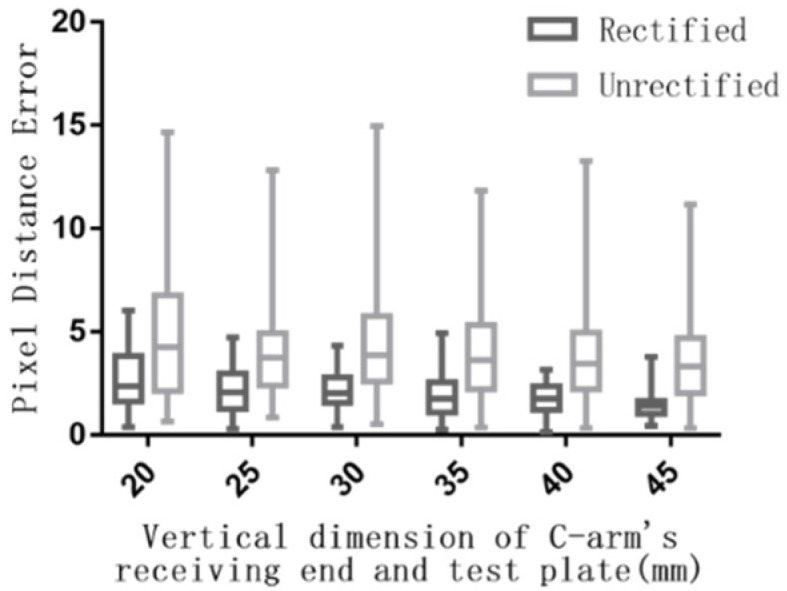
Error statistics of rectified and unrectified X-ray image.

Figure 9a showed the test plate for testing the results of image rectification and Figure 9b was the raw X-Ray image with obvious distortion. The image was significantly rectified, especially at edge of it in Figure 9c. Most errors of grid points were smaller on rectified image than on raw image (Figure 10). The mean error of each raw image was about twice as much as that of rectified image, and the max error of each raw image was about two and half times as much as that of rectified image at certain vertical distance between the plate and the receiving end of C-arm in Figure 11. The max error of raw images was 13.26, which was 3.47 times as much as that of rectified image. The max of the mean errors of all rectified images was 2.67. Besides, Figure 11 showed that the errors decreased with the increase of the vertical dimension between test plate and receiving end in general. 

### 3.2. Experiment of Calibration Accuracy

**Figure 12:**
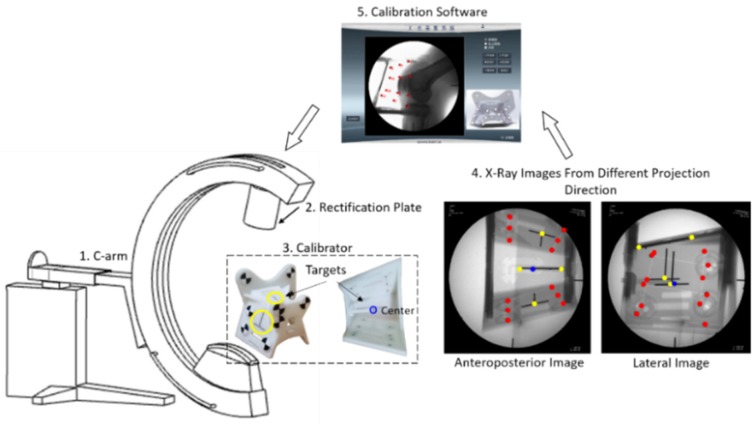
Structure of C-arm calibration experiment: the target at the center of the calibrator was marked in blue; other targets on the surface of the calibrator were marked in yellow; fiducial points were marked in red.

**Figure 13:**
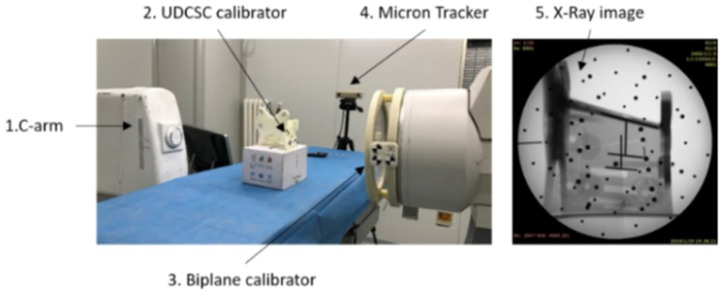
Comparison between UDCSC calibrator and biplane calibrator.

**Figure 14:**
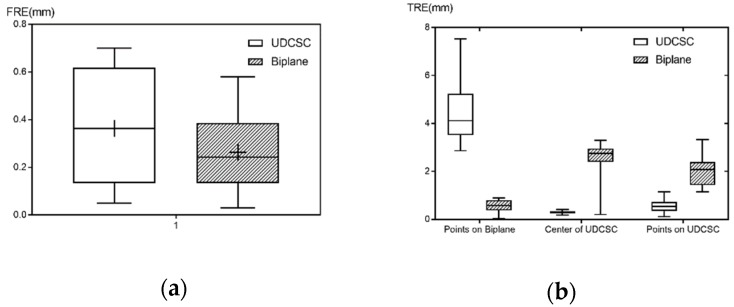
The errors of targets on calibrator.

Figure 12 showed that the calibration experiment platform consisted of C-arm (DG3310, Huadong Electronics, China), rectification plate, calibrator and our own calibration software. Anteroposterior and lateral X-Ray images captured by C-arm, were transferred to the software and corrected. Parameters of C-arm were calculated by the software. We designed 5 target points on the calibrator (Figure 12): one steel ball with a diameter of 3 mm were at the center of the calibrator; two targets, the cross of two steel lines (2 mm), were on the left and right surfaces of the calibrator; two targets, at the ends of a steel line (2 mm), were on the upper surface of the calibrator. Comparison of the calibration accuracy between UDCSC calibrator and biplane calibrator was conducted (Figure 13). Projections of calibrator points on UDCSC calibrator and biplane calibrator were recorded at the same time. Ten tests were conducted on anteroposterior projection and lateral projection respectively. FRE and TRE of each calibrator were calculated. Errors of calibrator points of each calibrator were used to calculate FRE. Target points, calibrator points on each calibrator were used to calculate TRE (Table 1). Points on the surface of UDCSC, including 12 calibrator points and 4 target points were calculated as one group. Figure 14 showed that the TRE of the entire calibrator was 0.41 mm, with 0.30 mm at the center of the calibrator.

### 3.3. Navigation and Positioning System for ACL Reconstruction

**Figure 15:**
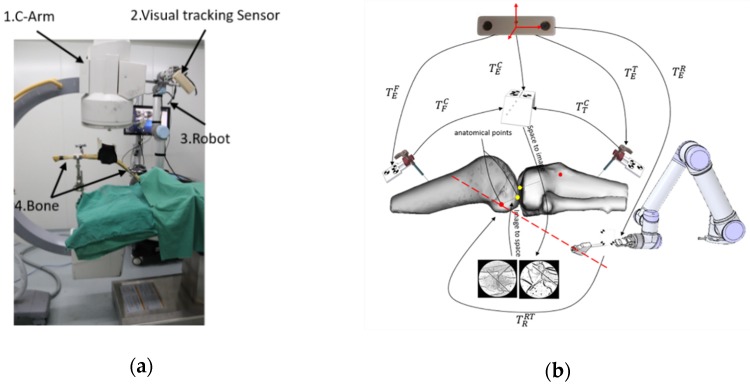
Experiment platform of navigation and positioning system: (**a**) Navigation System, which included C-Arm, visual tracking sensor, robot and bones; (**b**) Spatial transformation, $T_{E}^{F}$ represented transformation matrix of femur space and world coordinate system, $T_{F}^{C}$ represented transformation matrix of calibrator and femur space, $T_{E}^{C}$ represented transformation matrix of calibrator and world coordinate system, $T_{T}^{C}$ represented transformation matrix of calibrator and tibia space, $T_{E}^{T}$ represented transformation matrix of tibia space and world coordinate system, $T_{E}^{R}$ represented transformation matrix of tool coordinate system of robot and world coordinate system, $T_{R}^{RT}$ represented transformation matrix of reconstruction target and tool coordinate system of robot. Yellow points were end points of reconstruction tunnels, and red points were start points of reconstruction tunnels.

**Figure 16:**
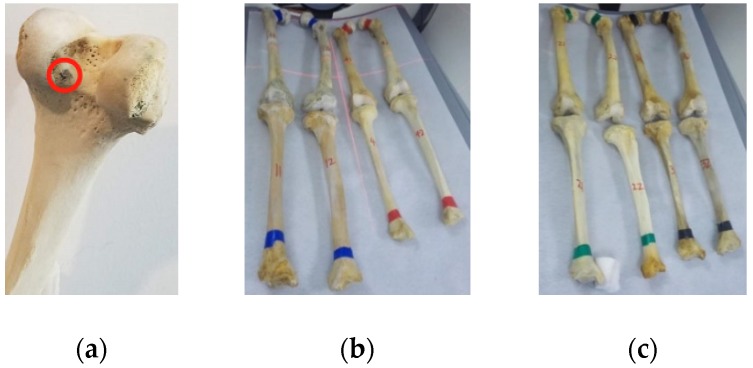
Dry bones for ACL navigation system: (**a**) Target on the femur: steel ball, marked with red circle, was embedded at both ends of the tunnels; (**b**) 2 pairs of dry cadaver femur and tibia (No.1 and No.4); (**c**) 2 pairs of dry cadaver femur and tibia (No.2 and No.3).

As shown on Figure 15a we developed a navigation and positioning system, which included C-Arm, Micron Tracker (Claron Technology Inc., Canada), UR5 Robotic arm (Universal Robot, Denmark), rectification plate, calibrator, bones with marked fixtures and a software. Specific black-white markers, which could be tracked through visual sensor, were on femur, tibia, calibrator (Figure 8d) and robot. The image rectification plate was fixed on the receiving end of the C-arm. The spaces of femur, tibia, robot, calibrator, operative target were unified into visual coordinate system through visual sensor (Figure 15b). Steel balls, with the diameter of 3 mm, were embedded at both ends of the tunnels (Figure 16a). They were start points and end points of tunnels for ACL reconstruction, which were also the targets of the positioning system. 2D-3D registration of normal navigation system was not needed in our tests. The software, developed by C++ ran on Windows OS, and the user interface of it was based on QT framework. OPENCV was used for image processing and DCMTK (Offis, Germany) provided an interface for DICOM protocol. The navigation and positioning system could construct the mapping between intraoperative image space and surgical space.

During surgery, positions of markers on the calibrator were recorded at the same time when the X-ray images were captured by C-arm. Two X-ray images at anteroposterior and lateral projection were used for calibration of C-arm. They were corrected by the method in Section 2.1 and intrinsic and extrinsic parameters of C-arm were calculated by the method in Section 2.2. The position information of planned tunnels, femur, tibia, and robot were converted to movements of the robot according to transformations in Figure 15b and the robot was ordered to move to the planned tunnel.

World coordinates of start points and end points of femurs and tibias $P_{C}^{FS}$, $P_{C}^{FE}$, $P_{C}^{TS}$, $P_{C}^{TE}$ could be calculated by a back projection model in Section 2.2. If $P_{1}$ and $P_{2}$ were two points on the line of instrument at the end of robot arm, world coordinates of which were known, the pixel position of them in every X-ray image at different projection directions could be calculated according to Equation (6). Straight line joined by the two points in X-ray image could be calculated and displayed on the user interface of the software. The relationship between the points of tunnels and feature-fixation holder of the bone, and the relationship between the points of tunnels and the robot could be expressed as follows:

(16) $$\begin{array}{ll} P_{F}^{\mathrm{FS}}{=(T}_{E}^{F}{)}^{-1}T_{E}^{C}P_{C}^{\mathrm{FS}} & P_{R}^{\mathrm{FS}}{=(T}_{E}^{R}{)}^{-1}T_{E}^{F}P_{F}^{\mathrm{FS}}\\ P_{F}^{\mathrm{FE}}{=(T}_{E}^{F}{)}^{-1}T_{E}^{C}P_{C}^{\mathrm{FE}} & P_{R}^{\mathrm{FE}}{=(T}_{E}^{R}{)}^{-1}T_{E}^{F}P_{F}^{\mathrm{FE}}\\ P_{T}^{\mathrm{TS}}{=(T}_{E}^{T}{)}^{-1}T_{E}^{C}P_{C}^{\mathrm{TS}} & P_{R}^{\mathrm{TS}}{=(T}_{E}^{R}{)}^{-1}T_{E}^{T}P_{C}^{\mathrm{TS}}\\ P_{F}^{\mathrm{TE}}{=(T}_{E}^{T}{)}^{-1}T_{E}^{C}P_{C}^{\mathrm{TE}} & P_{R}^{\mathrm{TE}}{=(T}_{E}^{R}{)}^{-1}T_{E}^{T}P_{T}^{\mathrm{TE}} \end{array}$$

**Figure 17:**
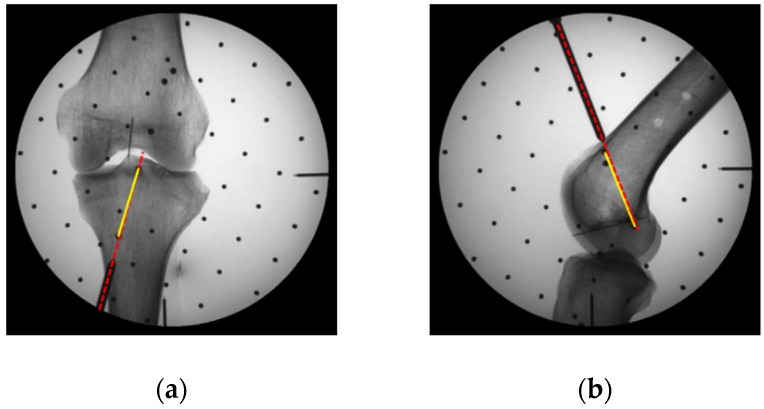
X-Ray images of positioning of the tunnels, planning tunnels through 2 steel balls was marked in yellow line and positioning tunnel was marked in red line: (**a**) Tibia planning tunnel; (**b**) Femoral planning tunnel.

If ${\mathrm{Err}}_{\mathrm{AS}}$ and ${\mathrm{Err}}_{\mathrm{LS}}$ were the pixel distance of start point to the navigation line (Figure 17), ${\mathrm{Err}}_{\mathrm{AE}}$ and ${\mathrm{Err}}_{\mathrm{LE}}$ were the pixel distance of end point to the navigation line (Figure 17), then the positioning error of start points and end points would be expressed as follows:

(17) $$\begin{array}{cc} {\mathrm{Err}}_{S}=\sqrt{{\mathrm{Err}}_{\mathrm{AS}}^{2}{+\mathrm{Err}}_{\mathrm{LS}}^{2}} & {\mathrm{Err}}_{E}=\sqrt{{\mathrm{Err}}_{\mathrm{AE}}^{2}{+\mathrm{Err}}_{\mathrm{LE}}^{2}} \end{array}$$

We performed navigation and positioning experiments on 4 pairs of dry cadaver femur and tibia (Figure 16b,c). Table 2 showed that the mean positioning errors of the start points and the end points were 0.81 mm and 0.88 mm, which means that the positioning tunnels were close to the planning tunnels through 2 steel balls (Figure 17).

## 4. Discussion and Conclusions

Image distortion and the errors of the camera calibration had a significant impact on the accuracy of navigation system [16]. A polynomial fitting-based global correction method was applied to rectify the raw X-Ray images in this paper. It was significant that the image rectification would result in smaller errors. 

We studied relationship between fiducial points and calibration errors and drew similar conclusions with Manning Wang [28] that the performance of FRE and TRE were not consistent. Although TRE decreased with the increase of the number of fiducial points, the decline was slow after 10. The performance of FRE were best at a number of 12. Besides, our study showed that TRE decreased with the increase of the uniformity of fiducial points in general. A calibrator with 12 points uniformly distributed conical helically was applied for online calibration of C-arm. However, as FREs range from 0.68 to 0.8 (Figure 5), better TRE can be achieved at a small cost of a slightly increasing of FRE by slightly increasing number of calibration points, if the uniformity of calibrator are slightly changed.

Comparison of calibration accuracy of different calibrators of UDCSC calibrator and Biplane calibrator were conducted. As was shown in Table 1, FRE of UDCSC and Biplane varied little. But TRE of UDCSC and Biplane were quite different with each other. When points on Biplane were considered as targets, the mean value of TRE of UDCSC was 3.30 mm, with a max value of 7.53 mm and a min value of 4.50 mm, and the mean value of TRE of Biplane were 0.58 mm, with a max value of 0.89 mm and a min value of 0.20 mm. While when points on UDCSC were considered as targets, the mean value of TRE of UDCSC was 0.91 mm, with a max value of 1.15 mm and a min value of 0.58 mm, and the mean value of TRE of Biplane was 2.30 mm, with a max value of 3.33 mm and a min value of 1.65 mm. And when five target points on UDCSC were considered as targets, the mean value of TRE of UDCSC was 0.38 mm, with a max value of 1.08 mm and a min value of 0.16 mm, and the mean value of TRE of Biplane was 2.03 mm, with a max value of 3.08 mm and a min value of 1.43 mm. As Biplane was fixed on the C-arm, calibrator points on Biplane were far away from UDCSC. Therefore, TRE of UDCSC was large when points on Biplane were considered as targets. Similarly TRE of Biplane waFas large when points on UDCSC were considered as targets, as targets were far away from Biplane. When targets were close to the space of calibrator, TRE of both UDCSC and Biplane were small. TRE decreased with the decrease of the distance between the target point and the center of calibration points in both simulation analysis and accuracy experiments. Surgical targets were far away from Biplane commonly, and close to the space of UDCSC. Overall, TRE of UDCSC at surgical space were smaller than that of Biplane. TRE of the space of the calibrator is 0.38 mm, which meets the positioning requirements of general orthopedic surgery. The calibration phantom, which could cover the operation targets on the knee joint, might be an attractive option for ACL reconstruction.

We developed a navigation system and performed positioning experiments on 4 pairs of dry cadaver femur and tibia. According to Parkinson [2], if the tunnel was outside the anatomic zone, then it was classified as non-anatomic and mode tunnel size was about 8 mm. In the research of Achtnich [3], the mean distance of the center of the tibial tunnel to the anterior cortex was 42.3% (±10.4) relative to the total sagittal diameter of the tibia. Jonathan [36] suggested that a 2-mm bridge of bone between the tunnel wall and the articular margin on the low (anatomically posterior) aspect of the notch should be leaved. The positioning accuracy of ACL reconstruction navigation system should be less than 2 mm. The positioning error of specimen experiments in Section 3.2, with a mean value of 0.85 mm, was a little higher than that of the calibration experiments in Section 3.3, but still sufficient for the ACL reconstruction surgery. The visual tracking camera (Micron Tracker) provided calibration accuracy of 0.2 mm RMS at depths of 40-100 cm. Transformation matrices of the tunnels of femur and tibia to the calibrator were calculated through the visual tracking system, and were not like that of the target points to the calibrator, which were given according to the design model. While the results of visual tracking camera might be influenced by the ambient light, NDI Polaris system, using IR markers, dose not suffer from this problem. It might be a preferred tracking system to increase the accuracy of navigation system. In addition, drilling or other operations could cause jitters of the platform and the fixation of markers on the bones were not completely rigid.

We embedded steel balls at both ends of the tunnels in the navigation experiments, to make sure that targets were clear on the X-Ray images. So that we did not perform the procedure of 2D-3D registration. But the surgical targets in actual surgery, the tunnels in ACL reconstruction, were planned in the 3D reconstruction models before operation. They were not distinct on the X-Ray images and should be determined through the registration of preoperative CT images and intraoperative images. In the future, we would focus on the 2D-3D registration and reducing the error caused by visual tracking system.

## Figures and Tables

**Table 1:** Comparison of calibration accuracy of different calibrators.

Error (mm)		UDCSC Calibrator				Biplane Calibrator			
		Max	Min	Mean	RMS	Max	Min	Mean	RMS
FRE		0.70	0.05	0.36	0.43	0.58	0.03	0.25	0.32
TRE	Points on Biplane	7.53	3.30	4.50	4.68	0.89	0.20	0.58	0.65
	Center of UDCSC	0.42	0.18	0.30	0.42	3.08	1.75	2.65	2.77
	Points on UDCSC	1.15	0.16	0.54	0.62	3.33	1.43	2.04	2.16

**Table 2:** Positioning Errors of the navigation system for ACL reconstruction.

Positioning Errors (mm)	Start Points	End Points
Max	1.55	1.89
Min	0.34	0.19
Mean	0.81	0.88
RMS	0.45	0.55
